# 

**Published:** 1909-07-10

**Authors:** 


### BOOKS RECEIVED.

City Press.
"The City of London Directory," 1909.

P. S. King and Son.
"The Problem of the Feeble-minded."

Charles Griffin and Co.
"Official Year-book of the Scientific and Learned Societies of Great Britain and Ireland."

Methuen and Co.
"Sister K," by Mabel Hart.

Hammond, Hammond and Company.
"Children of the Poor." By A. Davies Edwards, M.B., etc.

### THE GRADUATES' MEDICAL SCHOOLS.

DIARY FOR THE WEEK, JULY 12 to 17.

**LONDON SCHOOL OF CLINICAL MEDICINE, Seamen's Hospital, Greenwich, S.E.**

**At 2.15 p.m.**
**July 12,** Sir Dyce Duckworth, **Gouty Phlebitis.**
**At 3.15 p.m.**
**July 15,** Sir W. Bennett.

**THE POST-GRADUATE COLLEGE, West London Hospital, Hammersmith, W.**

**At 10 a.m.**
**July 12 and July 15,** Surgical Registrar, **Demonstration.**
**July 16,** Medical Registrar, **Demonstration.**
**At 12 noon.**
**July 12,** Dr. Bernstein, **Pathological Demonstration.**
**At 12.15 p.m.**
**July 13,** Dr. Pritchard, **Practical Medicine.**
**July 14 and July 17,** Dr. Grainger Stewart, **Practical Medicine.**
**At 5 p.m.**
**July 12,** Mr. Bidwell, **Practical Surgery—VI.**
**July 13,** Mr. Bidwell, **Operations for Gastric and Duodenal Hæmorrhage.**
**July 14,** Mr. Lloyd Williams, **Extraction of Teeth—When and How.**
**July 15,** Mr. Keetley, **Clinical Lecture.**
**July 16,** Mr. Armour, **Clinical Lecture.**

**MEDICAL GRADUATES' COLLEGE AND POLYCLINIC, 22 Chenies Street, W.C.**

**At 5.15 p.m.**
**July 12,** Mr. James Cantlie, **Tropical Diseases met with in England.**
**July 13,** Dr. Newton Pitt. **Cerebral Syphilis.**
**July 14,** Mr. Arbuthnot Lane, **The Surgical Treatment of Constipation.**
**July 15,** Dr. James Collier, **Disseminate Sclerosis.**

**NORTH-EAST LONDON POST-GRADUATE COLLEGE, Prince of Wales's General Hospital, Tottenham, N.**

**At 4.30 p.m.**
**July 13,** Dr. A. E. Giles, **The After-results of Ovariotomy.**

**NATIONAL HOSPITAL FOR THE PARALYSED AND EPILEPTIC, Queen Sq., Bloomsbury, W.C.**

**At 3.30 p.m.**
**July 13,** Dr. Gordon Holmes, **Familial Ataxia—Anomalous Cases.**

**THE HOSPITAL FOR SICK CHILDREN, Great Ormond Street, W.C.**

**At 4 p.m.**
**July 15,** Mr. Addison, **Syphilitic Disease of the Bones and Joints.**

**CENTRAL LONDON THROAT AND EAR HOSPITAL, Gray's Inn Road, W.C.**

**At 3.45 p.m.**
**July 13,** Mr. Gay French, **Tracheoscopy.**
**July 16,** Dr. Wyatt Wingrave, **Clinical Pathology.**

The following appointments to demonstratorships in the Medical School of St. Bartholomew's Hospital were made on July 1 : Operative Surgery, Mr. G. E. Gask, F.R.C.S. ; Anatomy, Mr. R. B. Etherington Smith, F.R.C.S., Mr. H. W. Wilson, F.R.C.S., and Mr. R. Foster Moore, F.R.C.S. ; Physiology, Dr. C. M. Hinds Howell ; Junior Demonstrators of Pathology, Dr. Harold Pritchard (medical), Mr. W. Girling Ball, F.R.C.S. (surgical) ; Pharmacology, Dr. F. A. Bainbridge ; Chemistry, Dr. K. S. Caldwell, B.Sc. ; Physics, Mr. F. Lloyd Hopwood, B.Sc. ; Biology, Dr. W. A. Cunnington, M.A. Cantab., Ph.D. Jena ; Midwifery, Mr. J. D. Barris, F.R.C.S.

THE HOSPITAL July 10, 1909.

***Name*** ..................................................

***Address*** ..................................................

..................................................

This Coupon must accompany manuscript or contributions intended for The Hospital.

